# Impacts of neurointerventional therapy combined with intravenous thrombolysis on neurological function, oxidative stress, and immune function in patients with acute ischemic stroke

**DOI:** 10.3389/fmed.2026.1721322

**Published:** 2026-02-05

**Authors:** Yun Wang, Yang Yang, Jingmin Zhou

**Affiliations:** Department of Neurology, Huai’an Hospital Affiliated to Yangzhou University (The Fifth People’s Hospital of Huai’an), Huai’an, Jiangsu, China

**Keywords:** acute ischemic stroke, immune function, intravenous thrombolysis, neurointerventional therapy, neurological function, oxidative stress

## Abstract

**Aim:**

This study aimed to assess the clinical efficacy of neurointerventional therapy plus intravenous thrombolysis in patients with acute ischemic stroke (AIS).

**Methods:**

We conducted a single-center retrospective analysis involving 120 AIS patients admitted to our hospital from January 2023 to December 2024. Based on their treatment plans, patients were categorized into an alteplase group (*n* = 55) and a combination group (*n* = 65). In both groups, all patients received standard intravenous thrombolysis with alteplase; in the combination group, this was followed by adjunct neurointerventional therapy, including intra-arterial urokinase infusion and, when indicated, mechanical thrombectomy. We compared neurological function scores, inflammatory factor levels, oxidative stress markers, immune function indicators, hemodynamic parameters, quality of life scores, and the total incidence of adverse reactions between the two groups.

**Results:**

After 3 months of treatment, the combination group demonstrated significantly lower NIHSS and mRS scores, as well as reduced levels of IL-6, TNF-α, and hs-CRP, compared to the alteplase group (*P* < 0.05). Additionally, the combination group exhibited higher SOD levels, lower MDA levels, elevated CD4^+^ counts and CD4^+^/CD8^+^ ratios, and decreased CD8^+^ levels (*P* < 0.05). Hemodynamically, the combination group had higher minimum cerebral blood flow volume and velocity, along with lower peripheral resistance in cerebral vessels (*P* < 0.05). Furthermore, the combination group achieved higher GQOLI-74 scores, indicating improved quality of life (*P* < 0.05). Notably, there was no significant difference in the total incidence of adverse reactions between the two groups (*P* > 0.05).

**Conclusion:**

Neurointerventional therapy plus intravenous thrombolysis can improve the neurological function, reduce inflammation and oxidative stress, enhance immune function, improve hemodynamic indicators, improve the quality of life and has good safety in the treatment of patients with AIS.

## Introduction

Acute ischemic stroke (AIS), as one of the more common cerebrovascular diseases in the nervous system, is mainly caused by narrowing or occlusion of the cerebral arteries, which leads to ischemia and hypoxia in the brain tissues (1). AIS belongs to the most common type of stroke, accounting for three-fourths of all strokes (2). It is currently the second leading cause of death as well as the third leading cause of disability all over the world (3). It is characterized by rapid onset, rapid changes in condition, high incidence, high mortality, high disability rate, and poor prognosis (4). Common sequelae after stroke include hemiplegia and speech disorders, which have a significant influence on patients’ quality of life and the living standards of their families, and have led to an increasing burden on national medical insurance (5). Therefore, the treatment and prevention of stroke have become the greatest challenge for society, economy, and health in our country.

Recent clinical and translational studies have highlighted that biological markers reflecting inflammation, oxidative stress, immune function and cerebral hemodynamics are closely related to the evolution and prognosis of AIS. Elevated inflammatory cytokines such as interleukin-6 (IL-6), tumor necrosis factor-α (TNF-α) and high-sensitivity C-reactive protein (hs-CRP), as well as oxidative stress indices such as malondialdehyde (MDA) and reduced superoxide dismutase (SOD) activity, have been associated with larger infarct volumes, early neurological deterioration and poorer functional outcomes, including in patients treated with intravenous thrombolysis (6, 7). Stroke-induced immune dysregulation, characterized by alterations in CD4+ and CD8+ T-cell counts and the CD4+/CD8+ ratio, is also linked to post-stroke infections and long-term outcomes (8). In parallel, non-invasive assessments of cerebral hemodynamics and health-related quality of life (HRQoL) after reperfusion have shown that more complete and sustained reperfusion after mechanical thrombectomy is associated with better perfusion parameters, functional independence and HRQoL (9). However, most available studies have examined these biomarkers or hemodynamic and HRQoL parameters in isolation or in cohorts treated with a single reperfusion modality, and comprehensive data integrating inflammatory, oxidative stress, immune, hemodynamic and HRQoL profiles in AIS patients remain limited.

In the treatment of AIS, intravenous thrombolysis and neurointerventional therapy are the two main treatment methods (10). Intravenous thrombolysis involves injecting thrombolytic drugs via the vein to dissolve the blood clot and restore blood flow; however, its time window is limited (usually within 4.5 h after the onset of the disease), and there are risks of bleeding and limitations in efficacy (11). Neurointerventional therapy reaches the thrombus site through the vascular internal route (such as intravascular thrombolysis and mechanical thrombectomy), enabling more precise restoration of blood flow (12). It is particularly effective for patients with large vessel occlusion (13). Since 2015, multiple randomized controlled trials and meta-analyses have shown that adding mechanical thrombectomy to standard intravenous alteplase markedly improves functional outcomes in patients with anterior-circulation large-vessel occlusion compared with intravenous thrombolysis alone, and has established mechanical thrombectomy as part of the standard of care (14, 15). Representative randomized trials and evidence syntheses comparing intravenous alteplase plus mechanical thrombectomy with either mechanical thrombectomy alone or alteplase alone have established endovascular thrombectomy as standard of care for anterior-circulation large-vessel occlusion and, overall, have not consistently shown non-inferiority of omitting intravenous alteplase, thereby clarifying the current role of “bridging therapy” with intravenous alteplase followed by thrombectomy in routine practice (16–20). Previous studies have pointed out that the therapeutic effect of using intravenous thrombolysis alone is limited, especially in patients who have exceeded the adaptation time window or have large vessel occlusion, where the thrombolysis effect is poor (21). Therefore, the comprehensive treatment method combining neurointerventional therapy and intravenous thrombolysis has gradually become a new research hotspot. This combined treatment aims to achieve a greater success rate and safety of vascular recanalization through multiple means working together, thereby improving the prognosis of patients (22). However, at present, this combined treatment protocol still requires more comprehensive clinical research data for AIS patients, especially regarding its impact on inflammatory responses, oxidative stress, immune function, cerebral hemodynamics, patient-reported quality of life and the incidence of adverse reactions.

Therefore, this study aimed to evaluate the clinical efficacy of this combined treatment regimen in patients with AIS, in order to improve the overall prognosis of the patients.

## Patients and methods

### Patients

We conducted a single-center retrospective analysis involving 120 AIS patients admitted to our hospital from January 2023 to December 2024. Inclusion criteria: (1) Met the diagnostic criteria of AIS and was confirmed through auxiliary examination methods such as CT and MRI; when feasible, non-invasive vascular imaging with CT angiography (CTA) or MR angiography (MRA) was also performed as part of the routine clinical assessment to screen for intracranial large-vessel occlusion or severe arterial stenosis; (2) All the patients had their first onset of the disease, and the time from symptom onset to reperfusion treatment met the time-window requirements (intravenous alteplase was initiated within 4.5 h of onset in all eligible patients, and for those in the combination group, arterial puncture for neurointerventional therapy was performed within 6 h of onset) (3) No relevant surgical contraindications. Exclusion criteria: (1) Patients who had experienced cerebral hemorrhage, abdominal or thoracic organ hemorrhage, or intracranial surgery within 6 months prior to enrollment; (2) Patients with liver or kidney dysfunction, or abnormal coagulation function; (3) Patients with contraindications for thrombolysis; (4) Patients with concurrent malignant tumors; (5) Patients with impaired consciousness or those unable to cooperate with treatment.

### Treatments

Patients were divided into an alteplase group and a combination group according to the reperfusion strategy actually implemented. Patients in the alteplase group received intravenous alteplase alone and did not undergo endovascular therapy. Patients in the combination group received the same intravenous alteplase regimen, followed by neurointerventional therapy. In this group, digital subtraction angiography identified intracranial large-vessel occlusion and/or severe arterial stenosis, and patients who were suitable for endovascular treatment underwent intra-arterial urokinase infusion and, when indicated, mechanical thrombectomy. The patients were administered alteplase (manufacturer: Boehringer Ingelheim Pharma GmbH & Co. KG) at a dose of 0.9 mg/kg, with a maximum dose not exceeding 90 mg. In the initial stage of treatment, 10% of the drug was dissolved in 10 mL of normal saline and injected into the peripheral vein group, and the remaining 90% of the drug was dissolved in 100 mL of normal saline and pumped into the vein using a venous pump over 1 h. After the treatment, a 24-h observation was conducted followed by a brain MRI, or a non-contrast CT scan when MRI was contraindicated or unavailable, to evaluate for intracranial hemorrhage. After there was no occurrence of cerebral hemorrhage, the patient was treated with aspirin (manufactured by Hefei Jiulian Pharmaceutical Co., Ltd.) orally, 0.5 g per day, once a day. The treatment was continued for 10 days, after which the dose was reduced to 0.1 g per day and was taken for 3 months. This aspirin regimen followed the institutional acute ischemic stroke protocol at our center, which is based on the domestic drug label and national clinical practice experience with short-term higher-dose aspirin in the early subacute phase of ischemic stroke followed by low-dose maintenance therapy. Patients in the combination group underwent neurointerventional therapy in addition to the intravenous alteplase regimen described above. After completion of intravenous alteplase, digital subtraction angiography was performed via femoral artery puncture to identify the occluded or severely stenosed intracranial arteries and assess collateral circulation in the ischemic region. In patients undergoing neurointerventional therapy, these angiographic findings served as the reference standard for confirming intracranial large-vessel occlusion and/or severe intracranial arterial stenosis and for guiding the subsequent endovascular procedure. If an occlusion or severe stenosis was confirmed, a solution containing 200,000 units of urokinase (manufactured by Livzon Pharmaceutical Group Inc.) diluted in 20 mL of 0.9% sodium chloride was slowly infused intra-arterially through a microcatheter positioned at the target lesion. When necessary, mechanical thrombectomy was performed according to the angiographic findings. Throughout the treatment, the patient’s vital signs were continuously and closely monitored. After completing the therapy, an arterial angiography was performed once more to assess the condition of the occluded blood vessels, and the treatment plan was adjusted based on the angiographic findings.

### Outcome and clinical assessment

Before and 3 months after treatment, the neurological function of patients was evaluated using the National Institutes of Health Stroke Scale (NIHSS) and the modified Rankin Scale (mRS) (23, 24). The NIHSS primarily comprised 11 dimensions, with each dimension scored on a scale from 0 to 42. A higher score on this scale indicated more severe neurological dysfunction. Meanwhile, the mRS was utilized to assess the recovery of patients’ neurological function, with scores ranging from 0 to 6; here, a higher score denoted poorer neurological function.

Before and 3 months following treatment, five ml of the patient’s fasting venous blood were obtained and centrifugated, and the serum was obtained. Serum interleukin-6 (IL-6), tumor necrosis factor-α (TNF-α), as well as high-sensitivity C-reactive protein (hs-CRP) were determined utilizing enzyme-linked immunosorbent assay (ELISA). The serum superoxide dismutase (SOD) and malondialdehyde (MDA) were respectively determined by chemiluminescence method and ELISA method. The levels of CD4^+^ and CD8^+^ in serum were determined using the FACSCalibur flow cytometer (from BD Company, United States), followed by calculating CD4^+^/CD8^+^.

Before and 3 months following treatment, Doppler ultrasound was used to measure the hemodynamic parameters of the patients. Examinations were performed by experienced sonographers using a standardized protocol. The hemodynamic parameters included the minimum blood flow volume, the minimum blood flow velocity, as well as the peripheral resistance of the cerebral vessels. Minimum blood flow volume was defined as the lowest mean volumetric blood flow (mL/min) recorded in the insonated intracranial artery during the cardiac cycle within the measurement period, and minimum blood flow velocity was defined as the lowest mean flow velocity (cm/s) over the same period. Peripheral resistance of the cerebral vessels was expressed as a Doppler-derived resistance index (RI), calculated as (peak systolic velocity − end-diastolic velocity)/peak systolic velocity, which is a commonly used index of cerebrovascular resistance in ultrasound-based hemodynamic studies.

Before and 3 months after treatment, the Generic Quality of Life Inventory-74 (GQOLI-74) was employed to evaluate patients’ quality of life (25). This inventory encompassed four key dimensions: psychological function, physical function, social function, and material living conditions. The total possible score was 100 points, with higher scores representing a better quality of life.

The total occurrence rate of adverse reactions including intracranial hemorrhage, gastrointestinal hemorrhage and skin and mucous membrane ecchymosis was statistically analyzed for patients. Intracranial hemorrhage was defined as any new hemorrhagic lesion within the cranial vault detected on CT or MRI during the acute post-treatment period, irrespective of whether it was symptomatic or asymptomatic.

### Statistical analysis

Statistical analyses were implemented utilizing GraphPad Prism 10.0. Quantitative data were expressed as the mean ± standard deviation (SD), while qualitative data were represented by counts or percentages. The normality of quantitative data was assessed utilizing the Kolmogorov-Smirnov test. For comparing differences between groups, categorical data were analyzed with the χ^2^ test, and continuous variables were evaluated using the independent samples Student’s *t*-test. Statistical significance was defined as a *p*-value less than 0.05.

## Results

### General data of patients

As presented in Table 1, no notable disparities were observed between the alteplase and combination groups concerning gender, age, duration from illness onset to hospital admission, as well as medical histories of cerebral infarction, hypertension, coronary heart disease, diabetes, smoking, as well as alcohol consumption (*P* > 0.05). Because all patients were treated according to the same institutional acute stroke protocol and within the guideline-recommended time window, the similar onset-to-admission times between the two groups suggest that there was no major imbalance in treatment timing. This suggested that the baseline demographic and clinical characteristics of the patients in both groups were comparable.

**TABLE 1:** General data of patients between the two groups.

Items	Alteplase group (*n* = 55)	Combination group (*n* = 65)	χ^2^/*t*-value	*P*-value
Gender			0.008	0.926
Male	30 (54.55)	36 (55.38)		
Female	25 (45.45)	29 (44.62)		
Age (years)	58.59 ± 6.42	58.65 ± 6.53	0.050	0.959
Time from onset of illness to admission to hospital (h)	4.47 ± 1.01	4.53 ± 1.03	0.320	0.748
History of cerebral infarction (%)	7 (12.73)	9 (13.85)	0.032	0.857
History of hypertension (%)	36 (65.45)	45 (69.23)	0.193	0.659
History of coronary heart disease (%)	8 (14.55)	10 (15.38)	0.016	0.897
History of diabetes (%)	10 (18.18)	13 (20.00)	0.063	0.800
History of smoking (%)	25 (45.45)	30 (46.15)	0.005	0.938
History of drinking (%)	27 (49.09)	32 (49.23)	0.000	0.988

### NIHSS and mRS scores

**FIGURE 1:**
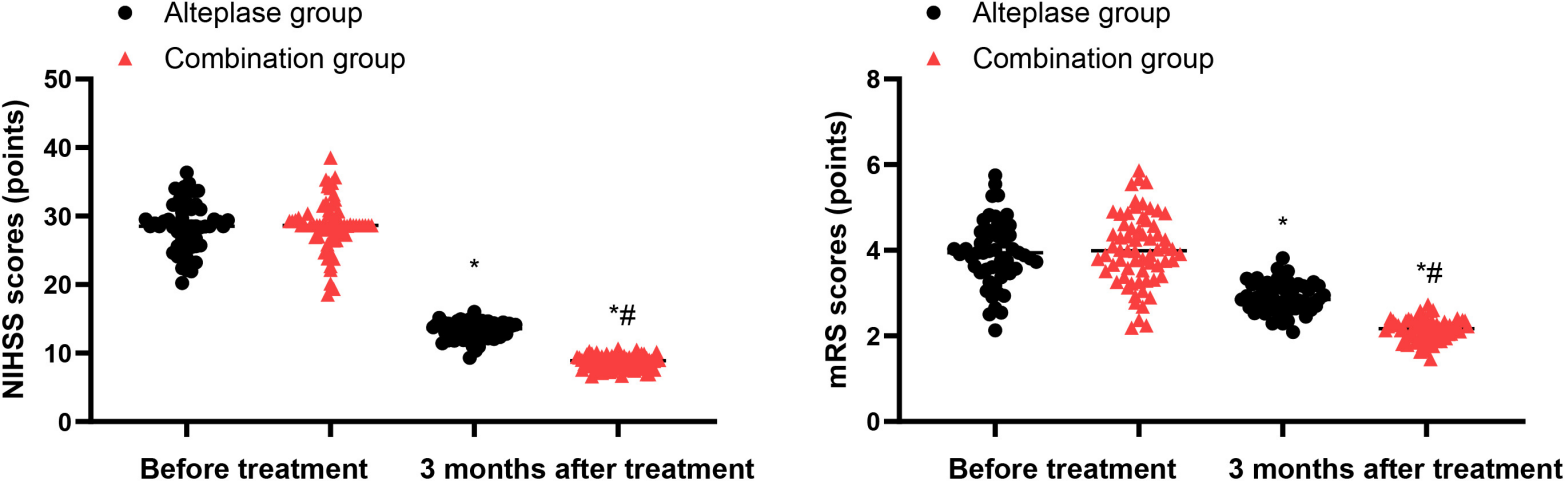
NIHSS and mRS scores between the two groups. *P < 0.05, vs. before treatment; ^#^*P* < 0.05, vs. alteplase group.

Prior to initiating the treatment, the NIHSS and mRS scores did not exhibit any differences between the alteplase and combination groups (*P* > 0.05). After undergoing 3 months of treatment, both groups demonstrated a reduction in NIHSS and mRS scores relative to their pre-treatment levels (*P* < 0.05). Furthermore, when comparing the two groups after the 3-month treatment period, the combination group displayed lower NIHSS and mRS scores than the alteplase group (*P* < 0.05) (Figure 1).

### Levels of inflammatory factors

**FIGURE 2:**
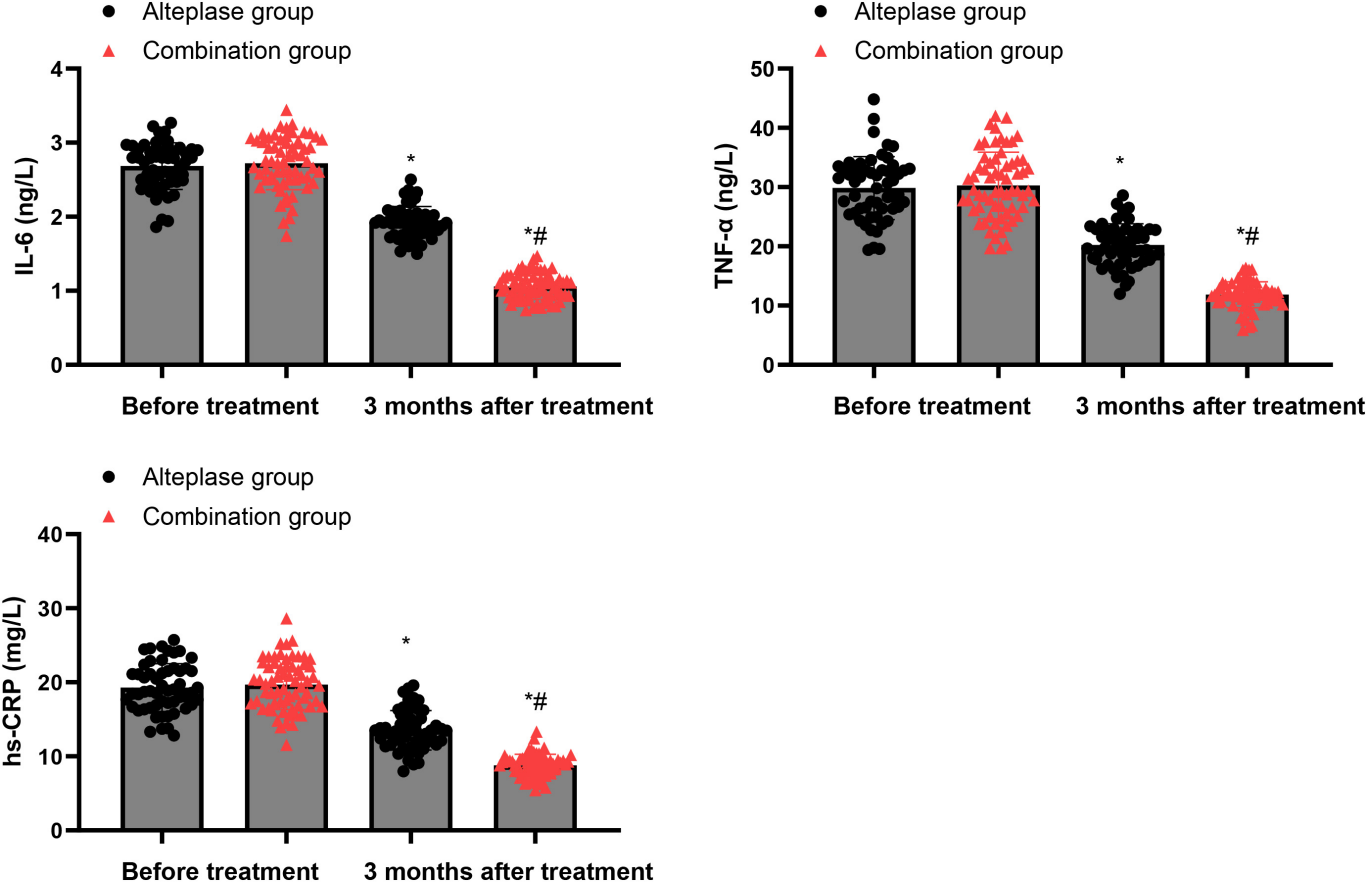
Levels of inflammatory factors between the two groups. *P < 0.05, vs. before treatment; ^#^*P* < 0.05, vs. alteplase group.

Prior to treatment, there were no differences in the serum levels of IL-6, TNF-α, and hs-CRP between the alteplase group and the combination group (*P* > 0.05). After completing 3 months of treatment, both groups showed a marked decrease in the levels of these inflammatory factors relative to their baseline values before treatment (*P* < 0.05). Moreover, when comparing the two groups after the 3-month treatment period, the combination group exhibited significantly lower levels of these inflammatory factors than the alteplase group (*P* < 0.05) (Figure 2).

### Levels of oxidative stress indicators

**FIGURE 3:**
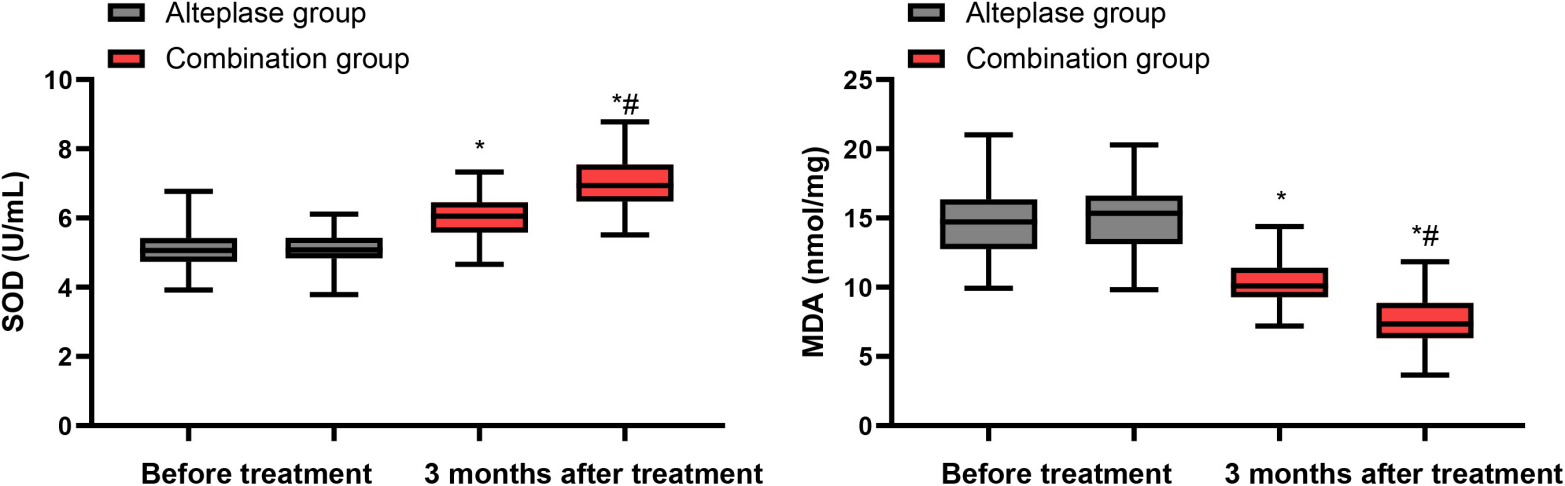
Levels of oxidative stress indicators between the two groups. *P < 0.05, vs. before treatment; ^#^*P* < 0.05, vs. alteplase group.

Before commencing the treatment, there were no notable disparities in SOD and MDA levels between the alteplase and combination groups (*P* > 0.05). After undergoing 3 months of treatment, both groups demonstrated a significant increase in SOD levels and a decrease in MDA levels relative to their pre-treatment values (*P* < 0.05). Furthermore, when the two groups were compared post-treatment, the combination group exhibited a higher SOD level and a lower MDA level than the alteplase group after the 3-month treatment period (*P* < 0.05) (Figure 3).

### Levels of immune function indicators

**FIGURE 4:**
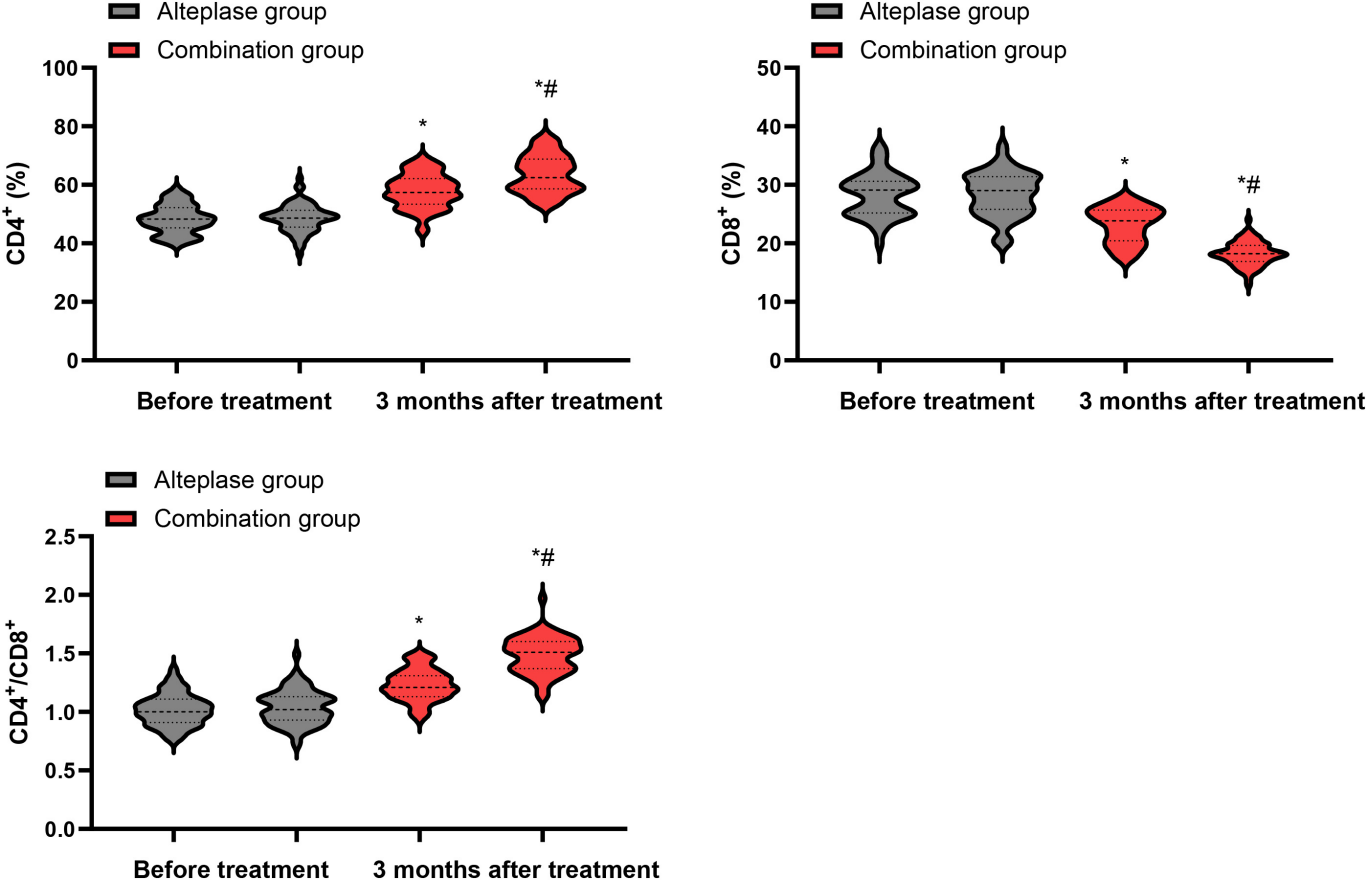
Levels of immune function indicators between the two groups. *P < 0.05, vs. before treatment; ^#^*P* < 0.05, vs. alteplase group.

Prior to the initiation of treatment, no differences were observed in the levels of CD4^+^, CD8^+^ and the CD4^+^/CD8^+^ ratio between the alteplase and combination groups (*P* > 0.05). After completing 3 months of treatment, both groups showed a notable increase in CD4^+^ levels and CD4^+^/CD8^+^ ratio, accompanied by a decrease in CD8^+^ levels relative to their pre-treatment values (*P* < 0.05). When comparing the two groups after the 3-month treatment period, the combination group exhibited higher levels of CD4^+^ levels and CD4^+^/CD8^+^ ratio, along with a lower CD8^+^ level, than the alteplase group (*P* < 0.05) (Figure 4).

### Hemodynamic parameters

**FIGURE 5:**
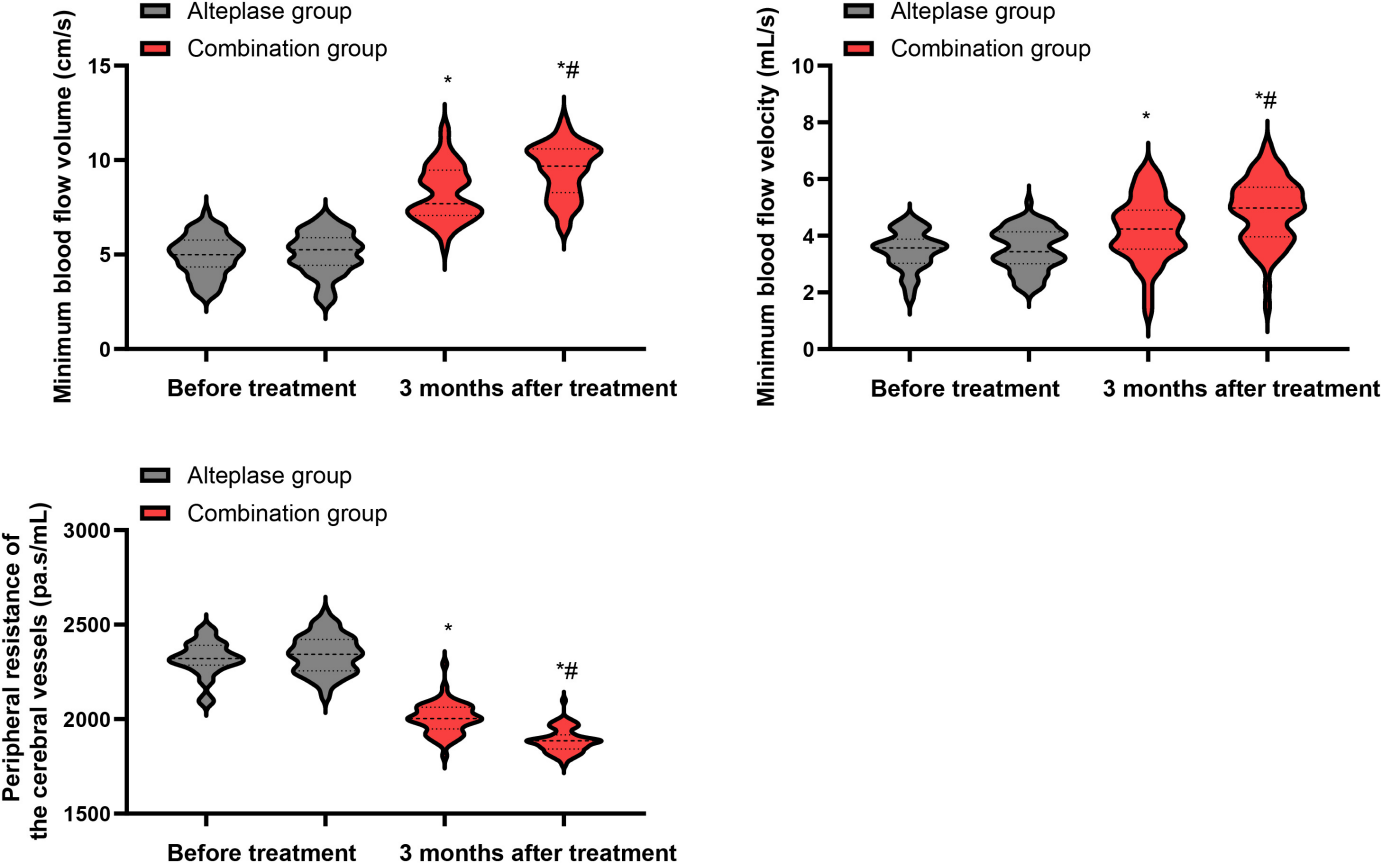
Hemodynamic parameters between the two groups. *P < 0.05, vs. before treatment; ^#^*P* < 0.05, vs. alteplase group.

Before treatment commenced, there were no statistically meaningful differences in the minimum cerebral blood flow volume, minimum cerebral blood flow velocity, as well as peripheral resistance of cerebral vessels between the alteplase and combination groups (*P* > 0.05). After undergoing 3 months of treatment, both groups demonstrated a significant increase in the minimum cerebral blood flow volume and minimum cerebral blood flow velocity, along with a notable decrease in the peripheral resistance of cerebral vessels relative to their pre-treatment values (*P* < 0.05). When the two groups were compared after the 3-month treatment period, the combination group exhibited higher minimum cerebral blood flow volume and minimum cerebral blood flow velocity, as well as lower peripheral resistance of cerebral vessels, than the alteplase group (*P* < 0.05) (Figure 5).

### GQOLI-74 scores

**FIGURE 6:**
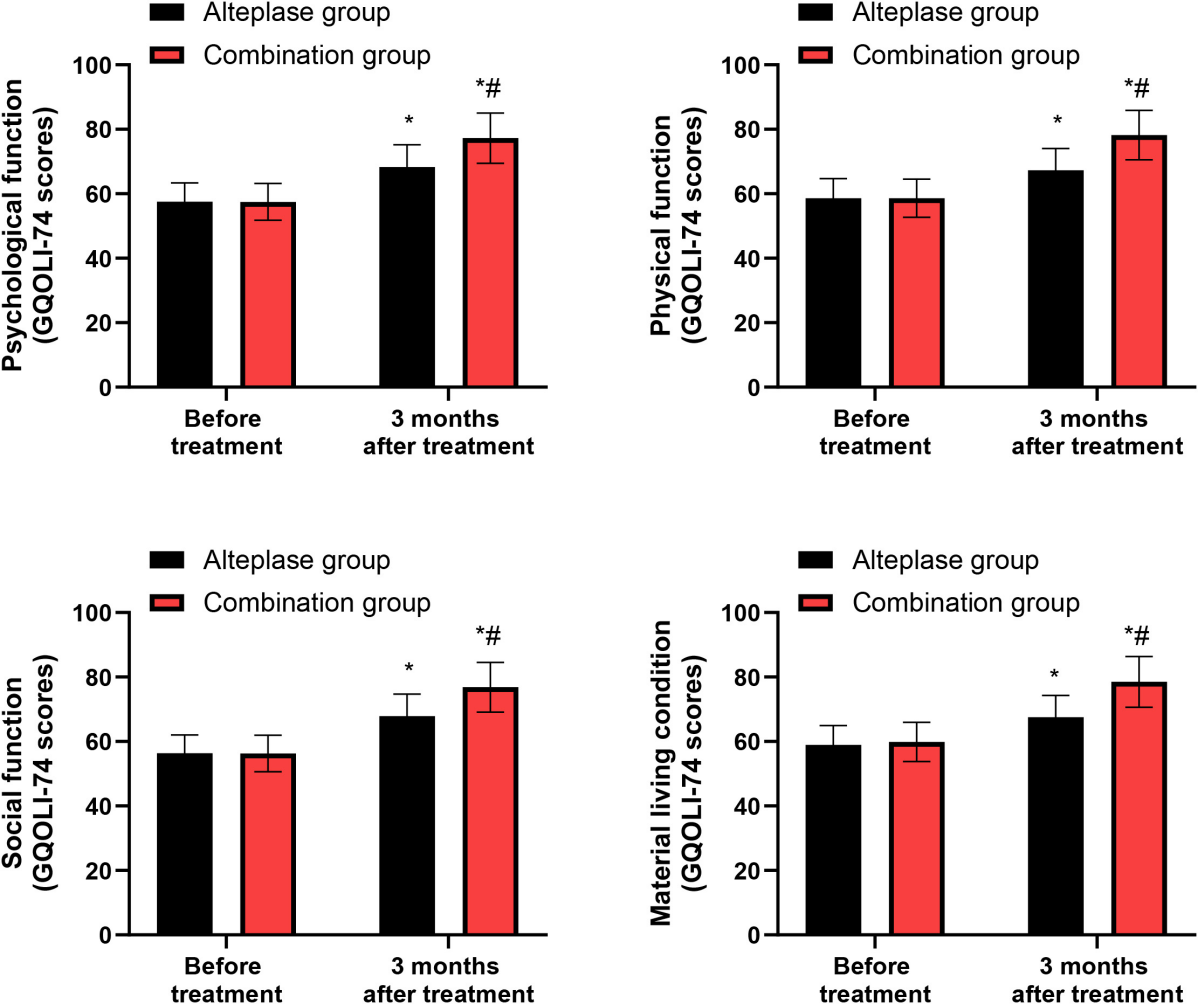
GQOLI-74 scores between the two groups. *P < 0.05, vs. before treatment; ^#^*P* < 0.05, vs. alteplase group.

Prior to treatment, no significant differences were evident in the GQOLI-74 scores pertaining to psychological function, physical function, social function, and material living conditions between the alteplase and combination groups (*P* > 0.05). After completing 3 months of treatment, both groups demonstrated a marked improvement in their GQOLI-74 scores across all measured domains relative to their pre-treatment scores (*P* < 0.05). When comparing the two groups post-treatment, the combination group exhibited higher GQOLI-74 scores than the alteplase group after the 3-month treatment period (*P* < 0.05) (Figure 6).

### Total occurrence rate of adverse reactions

As presented in Table 2, the total occurrence rate of adverse reactions did not differ significantly between the alteplase group and the combination group (*P* > 0.05), indicating that the combined treatment approach maintains a comparable safety profile.

**TABLE 2:** Total occurrence rate of adverse reactions between the two groups.

Groups	Cases	Intracranial hemorrhage	Gastrointestinal hemorrhage	Skin and mucous membrane ecchymosis	Total occurrence rate
Alteplase group	55	5 (9.09)	1 (1.82)	1 (1.82)	7 (12.73)
Combination group	65	4 (6.15)	2 (3.08)	2 (3.08)	8 (12.31)
χ^2^-value					0.004
*P*-value					0.944

Intracranial hemorrhage includes all radiologically confirmed intracranial hemorrhagic events (symptomatic and asymptomatic).

## Discussion

Intravenous thrombolysis therapy is a commonly used treatment method for AIS, which can significantly improve the cerebral blood perfusion of patients and thereby alleviate the damage caused by cerebral ischemia (26). However, this therapy has some significant limitations. Firstly, there are many contraindications for intravenous thrombolysis, including bleeding tendency and severe hypertension, which limit its application scope (27); secondly, during the treatment process, bleeding complications such as cerebral hemorrhage or gastrointestinal bleeding may occur, increasing the treatment risk (28); additionally, some patients may still experience recurrent embolism after treatment, resulting in poor treatment outcomes (29). Therefore, it is essential to implement other intervention measures on the basis of intravenous thrombolysis in order to further enhance the therapeutic effect (30). In the present study, the neurointerventional therapy protocol consisted of intra-arterial urokinase infusion via femoral artery access, with mechanical thrombectomy performed when indicated, on the basis of standard intravenous alteplase thrombolysis.

Our study assessed the clinical efficacy of neurointerventional therapy plus intravenous thrombolysis in patients with AIS. The results indicated that after undergoing 3 months of treatment, both groups exhibited a reduction in NIHSS and mRS scores compared to their pre-treatment levels. Furthermore, when comparing the two groups after the 3-month treatment period, the combination group displayed lower NIHSS and mRS scores than the alteplase group. The potential reasons for this difference are as follows: (1) Neurointerventional treatment can directly restore the blocked blood vessels through mechanical thrombectomy or angioplasty. This immediate vascular reconstruction effect complements the chemical thrombolysis effect of alteplase, effectively shortening the ischemic time of brain tissue (31). (2) The combined treatment strategy can restore cerebral blood flow perfusion through multiple pathways, which can save a larger penumbra area and thereby reduce neuronal apoptosis and secondary brain injury. (3) Early vascular recanalization may improve the metabolic microenvironment of the brain, enhance neural plasticity, and promote functional compensatory reconstruction. The synergistic action of these pathophysiological mechanisms may be the key reason for better neurological prognosis in the combined treatment group. Consistently, Li et al. suggested that the combination of neurointerventional therapy and intravenous thrombolysis not only effectively enhances the condition of affected blood vessels and aids in the recovery of impaired nerve function but also significantly lowers the complication rate in patients suffering from ischemic cerebrovascular disease (32).

AIS is a cerebrovascular circulatory disorder caused by multiple factors. Among them, inflammatory responses play a role throughout the entire process of AIS occurrence and development by promoting atherosclerosis of cerebral arteries, thrombosis, cerebral ischemia-reperfusion injury, and neuronal necrosis (33). IL-6 is an inflammatory factor secreted by lymphocytes, and its elevated level can promote the secretion and release of oxygen free radicals and excitatory amino acids, thereby inducing apoptosis of vascular endothelial cells (34). TNF-α is an inflammatory factor secreted by mononuclear macrophages, which can promote the secretion of IL-6 and other inflammatory factors and regulate the immune response of the body (35). hs-CRP is an acute-phase response protein secreted by liver cells. Under normal circumstances, its level is extremely low. When inflammatory factors are released in increased amounts, the level of hs-CRP rises sharply, and it is positively correlated with the degree of inflammatory response in the body (36). Consistent with these mechanisms, we observed that inflammatory markers decreased over time in both groups and were consistently lower in the combination group than in the alteplase group after 3 months of treatment, suggesting that combined therapy may more effectively attenuate post-stroke inflammatory activation. This is because combined therapy may achieve a multi-target synergy, in addition to dissolving blood clots, to further inhibit the activation of monocytes and macrophages and block the release pathways of inflammatory factors, thereby more thoroughly regulating the inflammatory response network; while monotherapy may only act on specific links, resulting in relatively limited effects on controlling inflammation.

During the pathological physiological process of the nervous system, various factors such as cerebral ischemia, hypoxia, stress and inflammatory responses all disrupt the dynamic balance of the body’s oxidation and antioxidant systems, leading to excessive generation of oxygen free radicals (37). At the same time, the body’s antioxidant capacity declines, which not only causes damage to neuronal mitochondria but also promotes the increase in vascular endothelial cell permeability (38). This is an important mechanism leading to neuronal apoptosis and neurological function impairment. MDA is an intermediate product of lipid peroxidation in cell membranes (39). SOD is a specific enzyme reflecting the body’s antioxidant capacity (40). In our cohort, SOD levels increased and MDA levels decreased over 3 months in both groups, with a more favorable oxidative profile in the combination group, supporting the notion that more complete reperfusion may enhance endogenous antioxidant defenses and limit lipid peroxidation. We believe that the reason why the combined treatment group demonstrated a superior antioxidant effect might be that it adopted a multi-target and multi-pathway intervention strategy, not only targeting the direct generation of oxygen free radicals, but also enhancing the overall antioxidant defense capacity of the body, thereby achieving a more comprehensive neuroprotective effect.

Patients with AIS are in a state of high metabolism due to factors such as dysphagia and hemiplegia. This can lead to increased intestinal permeability, gastrointestinal dysfunction, and malnutrition, thereby causing immune dysfunction (41). It not only increases the occurrence of complications such as infections, but also seriously affects the recovery of neurological function (42). In line with these concepts, we found that immune indices generally improved in both groups over 3 months, with more pronounced increases in CD4+ T-cell counts and CD4+/CD8+ ratio and a greater reduction in CD8+ T-cell counts in the combination group, suggesting a more favorable recovery of immune homeostasis. The reason for this difference might be that the combined treatment, through multi-pathways and multiple targets, exerts a more comprehensive regulatory effect on the patient’s immune system, promoting the activation of immune cells and effectively repressing the activity of immunosuppressive cells, thereby achieving a better recovery of immune function.

In our study, both groups showed improvements in cerebral blood flow parameters over time, and these changes were more favorable in the combination group, with higher minimum blood flow volume and velocity and lower peripheral resistance at 3 months, consistent with more complete macrovascular and microvascular reperfusion. This may be because all patients received intravenous alteplase, and those in the combination group additionally underwent intra-arterial urokinase administration and mechanical recanalization, which together can more effectively restore patency of the culprit vessels, improve microcirculation and collateral flow, increase arterial blood flow velocity, and reduce peripheral vascular resistance (43, 44). When used at an early stage in patients with stroke, timely reperfusion therapy can promote the recovery of ischemic neurons (45). Therefore, after treatment, the neurological deficits and symptoms of stroke in patients are significantly improved. Combined with interventional therapy, the blocked blood vessels can be further unblocked, and the treatment outcome can be improved.

These observations are in line with a broader body of evidence indicating that combining systemic rt-PA with endovascular strategies can optimize both macrovascular and microvascular reperfusion. Multiple randomized trials and meta-analyses comparing direct mechanical thrombectomy with “bridging therapy” using intravenous alteplase up to the time of thrombectomy have shown that endovascular thrombectomy on top of alteplase substantially improves functional outcomes relative to alteplase alone and that omission of intravenous alteplase has not consistently demonstrated clear non-inferiority (46, 47). In addition, phase II randomized data from the CHOICE trial and subsequent studies suggest that low-dose intra-arterial alteplase administered after angiographically successful thrombectomy may further increase the likelihood of excellent functional outcome without a major increase in symptomatic intracranial hemorrhage (5, 48). Although the present study used intra-arterial urokinase rather than alteplase, the principle of adjunct intra-arterial thrombolysis after or during mechanical recanalization is similar and may help to address residual thrombotic burden in distal vessels and the microcirculation, thereby contributing to the more favorable hemodynamic profile observed in the combination group.

Moreover, GQOLI-74 scores improved in both groups over 3 months and were higher in the combination group, indicating that the more favorable clinical and biological profiles were accompanied by better health-related quality of life. These results suggest that neurointerventional therapy combined with intravenous thrombolysis can improve the quality of life of AIS patients. Meanwhile, the total occurrence rate of adverse reactions did not differ significantly between the alteplase group and the combination group, indicating that the combined treatment approach maintains a comparable safety profile.

This study also has some limitations. First, it was a single-center retrospective analysis with a relatively small sample size, which may limit the generalizability of the findings. Second, because of its retrospective design, exact onset-to-needle and onset-to-puncture times were not systematically and uniformly recorded in the electronic database and therefore could not be retrieved for precise numerical analysis. Instead, we used the time from symptom onset to hospital admission, which did not differ significantly between the two groups, as a surrogate measure of treatment timing. Although all patients were treated within the guideline-recommended time window according to the institutional acute stroke protocol, the lack of detailed onset-to-treatment time data may limit a more refined assessment of the impact of treatment delays on clinical outcomes. Third, because of the retrospective design, the exact site of large-vessel occlusion or severe stenosis (for example, individual segments of the middle cerebral artery, posterior cerebral artery, or basilar artery) and the baseline infarct core (e.g., Alberta Stroke Program Early CT Score or quantitative lesion volume) were not systematically coded in the electronic medical records and therefore could not be reliably retrieved for artery-specific or core-size-specific analyses. These limitations may reduce the precision of our estimates of treatment effect and highlight the need for future prospective studies with more detailed imaging and outcome documentation. Furthermore, retrospective identification of adverse events may underestimate their true frequency, and in-hospital and post-discharge mortality were not analyzed as separate prespecified endpoints; the status of patients with mRS score 6 (death) at 3 months is now explicitly indicated in the revised figure, but more detailed mortality analyses were beyond the scope of the present study. In addition, the main clinical and biological outcomes were assessed at 3 months, which is consistent with the standard 90-day endpoint used in most acute ischemic stroke trials but does not capture longer-term recovery or late adverse events; future prospective studies should therefore incorporate systematic 6- and 12-month follow-up to more fully evaluate the durability and long-term safety of the combined therapy.

## Conclusion

Neurointerventional therapy combined with intravenous thrombolysis can improve the neurological function, reduce inflammation and oxidative stress, enhance immune function, improve hemodynamic indicators, improve the quality of life and has good safety in the treatment of AIS patients.

## Data Availability

The original contributions presented in this study are included in this article/supplementary material, further inquiries can be directed to the corresponding author.
